# Hospital-onset methicillin-resistant *Staphylococcus aureus* bloodstream infections within tertiary and community hospitals and implications for prevention

**DOI:** 10.1017/ice.2025.10370

**Published:** 2026-03

**Authors:** Harjot Kaur Singh, Barbara Ross, Joyce Hannah, Serena Ting, Chloe Teasdale, Xiao Wang, Margaret Quinn, David Calfee, Matthew Simon, Heidi Torres, Karen Acker, Harold Horowitz, Tina Wang, Nuwan Gunawardhana, Robin Golderg, Yolima Salazar, Nishant Prasad, Nadia Jagnatnarain, Candace Johnson, David Kuang, Adam Gouveia, Yoko Furuya, Karen Westervelt, Lisa Saiman

**Affiliations:** 1 Department of Medicine, Weill Cornell Medicine, New York, NY, USA; 2 Department of Infection Prevention & Control, NewYork-Presbyterian Hospital, New York, NY, USA; 3 Columbia University Mailman School of Public Health, New York, NY, USA; 4 The City University of New York School of Public Health and Health Policy, New York, NY, USA; 5 Department of Pediatrics, Weill Cornell Medicine, New York, NY, USA; 6 NewYork-Presbyterian Brooklyn Methodist Hospital, New York, NY, USA; 7 Department of Medicine, Columbia University Irving Medical Center, New York, NY, USA; 8 NewYork-Presbyterian Westchester Hospital, Bronxville, NY, USA; 9 NewYork-Presbyterian Queens Hospital, New York, NY, USA; 10 NewYork-Presbyterian Hudson Valley Hospital, Cortlandt, NY, USA; 11 Department of Pediatrics, Columbia University Irving Medical Center, New York, NY, USA

## Abstract

**Background:**

An improved understanding of the epidemiology of hospital-onset methicillin-resistant *Staphylococcus aureus* bloodstream infection (HO-MRSA BSI) could inform future prevention strategies for HO-MRSA BSI.

**Methods:**

We performed a retrospective cohort study of HO-MRSA BSI reported to NHSN from 2020–2023 at a system of 9 acute care hospitals located in New York City. The primary outcome was to describe the demographic and clinical characteristics of patients with HO-MRSA BSI. Secondary outcomes included comparisons of tertiary (TH) and community (CH) hospitals, standardized infection ratio (SIR) and rates per 10,000 patient-discharges, presumptive potential infectious sources, and mortality.

**Results:**

Between 2020 and 2023, 222 patients had HO-MRSA BSI. Their median age was 65 years, 139 (63%) were male, 92 (41%) had central lines, 89 (40%) were in ICUs, and 63 (28%) were on a ventilator. These characteristics were similar across the 176 (79%) patients in TH and the 46 (21%) patients in CH. SIRs were similar across each year of the study (with cumulative SIRs of 0.815 overall, 1.412 [CH] and 0.732 [TH]). Overall HO-MRSA BSI rates ranged from 2.58–3.53 per 10,000 patient-discharges. The most common sources of HO-MRSA BSI were pneumonia (41%), SSTIs (17%), CLABSIs (13%), and PIV catheter-related issues (9%). The all-cause mortality rate was 35%.

**Discussion:**

The unchanged HO-MRSA BSI SIRs in this study support the need for additional interventions that focus on prevention of the primary sources of MRSA infections. Ongoing systematic surveillance of the primary sources of HO-MRSA BSI should be implemented to inform and monitor best practices for prevention.

## Introduction

Since 2013, the Centers for Medicare and Medicaid Services (CMS) have required surveillance for laboratory-identified (LabID) hospital-onset methicillin-resistant *Staphylococcus aureus* bloodstream infection (HO-MRSA BSI) which is reported to the Centers for Disease Control and Prevention’s (CDC) National Healthcare Safety Network (NHSN).^1,2^ Since surveillance was initiated, the incidence of HO-MRSA BSI has not substantially changed, potentially due in part to reliance on central line-associated bloodstream infection (CLABSI) prevention bundles as the main prevention strategy for HO-MRSA BSI.^3,4^ However, many HO-MRSA BSI are not CLABSIs and less than half of CLABSI’s are due to MRSA.^5^ Thus, an improved understanding of the epidemiology of HO-MRSA BSI could inform prevention efforts focused on non-CLABSI sources of HO-MRSA BSI. The primary objective of this study was to describe the demographic and selected clinical characteristics of patients with HO-MRSA BSI using data between 2020 and 2023 from our multi-hospital healthcare system. Secondary objectives included [1] comparisons of these characteristics among patients with HO-MRSA BSI at tertiary versus community hospitals, [2] trend standardized infection ratios (SIRs) and rates per 10,000 discharges, [3] determine the primary sources of MRSA infections leading to HO-MRSA BSI, and [4] assess all-cause mortality and length of stay in patients with HO-MRSA BSI in tertiary (TH) versus community (CH) hospitals.

## Methods

### Study design, setting, and patient population

We performed a retrospective cohort study of LabID HO-MRSA BSI reported to NHSN between 2020 and 2023 at a healthcare system of 9 acute care hospitals located in the New York City metropolitan area to determine common primary sources of HO-MRSA BSI to inform prevention strategies. The healthcare system consists of 4 community and 5 tertiary hospitals, including 1 freestanding children’s tertiary hospital. As per our system’s classifications, hospitals were categorized as tertiary versus community based on the former’s provision of specialized complex care and availability of training programs. The institutional review boards responsible for the 9 hospitals reviewed this study and deemed it Non-Human Subjects Research.

### Infection prevention & control practices

The healthcare system shares the same Department of Infection Prevention and Control (IP&C) and electronic medical record (EMR) system, Epic^®^ (Verona, WI). MRSA surveillance and decolonization are performed at providers’ discretion, except for infants in the neonatal intensive care unit (ICU) at the children’s hospital or children undergoing cardiac surgery for whom routine surveillance and decolonization for MRSA were performed. In addition, an MRSA decolonization policy was available but not routinely recommended in our network. Patients with MRSA colonization or infection are placed on contact precautions (gloves and gowns) which are discontinued if two PCR swabs from the anterior nares are negative or discontinued after one year if no further PCR tests or cultures are MRSA-positive. During the study period, there were no standardized tracking of ventilator- or non-ventilator-associated pneumonia prevention bundles. Chlorhexidine gluconate (CHG) bathing treatment was recommended for all patients with central lines and all patients in the ICU; with system-wide tracking available in 2023.

### Identifying HO-MRSA BSI

A report of MRSA-positive blood cultures in hospitalized patients was generated weekly to identify new, unique MRSA-positive blood cultures and hospital-onset cases were electronically transferred to NHSN using NHSN case definitions.^2^ In 2020, HO-MRSA BSI became one of the healthcare system’s top 10 quality metrics. After transition to Epic^®^, IP&C data analysts tracked MRSA-positive cultures entered into NHSN and notified the responsible hospital epidemiologists of newly identified HO-MRSA BSI cases to assess primary source(s) of infection leading to HO-MRSA BSI on a weekly basis. In 2021, a near real-time review process was introduced to standardize data collection and systematically identify primary sources of infection. No new standardized interventions were implemented for HO-MRSA BSI during the study period.

### Data collection and outcomes

For each episode of HO-MRSA BSI, demographic and selected clinical characteristics, including a positive SARS-CoV-2 test within the 30 days prior to HO-MRSA BSI, were extracted from the EMR. Results of MRSA cultures or anterior nares PCR tests in the year prior to HO-MRSA BSI were collected to explore the potential impact of implementing MRSA decolonization strategies.

HO-MRSA BSI was defined by NHSN and included all cases of MRSA bacteremia that developed on or after day 4 of hospitalization.^2^ To assess primary sources of infection, NHSN case definitions were used for CLABSIs, catheter-associated urinary tract infections (CAUTIs), and surgical site infections (SSIs).^2^ The hospital epidemiologists, all board-certified infectious disease physicians, identified potential infectious sources using clinical interpretation and judgment. If the primary source could not be determined, the source was characterized as unknown. A HO-MRSA BSI episode could be attributed to more than one source and patients could have more than one episode. HKS and LS selectively reviewed the attributions of hospital epidemiologists for primary sources of infection to improve consistency. If there were discrepancies, HKS and LS discussed cases to reach consensus.

Outcomes included SIR and rates of HO-MRSA BSI per 10,000 patient-discharges at tertiary versus community hospitals each year, as well as the in-hospital and 30-day all-cause mortality rates overall length of hospital stay, and length of stay until first MRSA-positive blood culture.

### Data analysis

Demographic and clinical characteristics of patients with HO-MRSA BSI were compared between tertiary and community hospitals using Wilcoxon Rank Sum test, χ^2^ test, and Fisher’s exact test using SAS software, version 9.4M7© 2020, SAS Institute Inc., Cary, NC, USA. For those patients with more than one episode in different years, analysis of demographic and clinical characteristics used relevant data for the first HO-MRSA BSI episode only. For comparisons across years, we used χ^2^ tests, Kruskal-Wallis for medians, and Fisher’s Exact tests when counts were small. SIRs were calculated using NHSN methodology.^6^ SIRs between all paired years and between hospital type were compared using NHSN statistical tools.^7^

HO-MRSA BSI rate comparisons were performed using χ^2^ tests, two years at a time. All potential sources of HO-MRSA BSI were counted from all episodes, including if more than one source was identified from a single episode. To analyze in-hospital all-cause mortality, 30-day all-cause mortality, and the number of days between HO-MRSA BSI and death for patients with more than one episode, data from the last HO-MRSA episode were used. To analyze the number of days from admission until HO-MRSA BSI diagnosis and the overall length of stay, the first HO-MRSA BSI episode was used. For mortality comparisons, we used Wilcoxon rank sum tests for medians, χ^2^ test, and fisher’s exact test. Length of stay was compared using Wilcoxon rank sum tests.

## Results

### Demographic and clinical characteristics

Between 2020 and 2023, 222 patients were diagnosed with 232 episodes of HO-MRSA BSI; 8 patients had 2 episodes, and 1 patient had 3 episodes. These 232 episodes represented 0.026–0.035% of annual patient discharges. The median age of patients was 65 years (IQR 50–76), 139 (63%) were male, 85 (38%) were White, 54 (24%) were Black, and 61 (27%) were Hispanic (Table 1). Overall, 92 (41%) patients had central lines, 89 (40%) were in the ICU, and 63 (28%) were on a ventilator. Within the 30 days prior to HO-MRSA BSI, 36 (18%) of 195 patients tested were SARS-CoV-2-positive. Within the one year prior to HO-MRSA BSI, 65 (73%) of 89 patients evaluated had a positive MRSA PCR and 64 (29%) of 222 patients had a clinical culture positive for MRSA. The demographic characteristics of patients with HO-MRSA BSI were similar for each year of the study period,

**Table 1.** Demographic and clinical characteristics of patients with hospital-onset methicillin-resistant *Staphylococcus aureus* bloodstream infection (HO-MRSA BSI) in tertiary versus community hospitals, 2020–2023

Characteristics	All patients *n* = 222^1^	Tertiary hospitals *n* = 176 (79%)	Community hospitals *n* = 46 (21%)	*P*-value
**Demographic characteristics**				
Age, years, median (IQR)	65 (50–76)	63 (46–75)	69 (54–77)	**0.04**
Age strata, years, *n* (%)				0.12
< 18	27 (12)	25 (14)	2 (4)	
18–64	83 (37)	68 (39)	15 (33)	
65–74	50 (23)	39 (22)	11 (24)	
≥ 75	62 (28)	44 (25)	18 (39)	
Sex, *n* (%)				0.92
Male	139 (63)	110 (63)	29 (63)	
Female	83 (37)	66 (38)	17 (37)	
Race, *n* (%)				
White	85 (38)	62 (35)	23 (50)	0.19
Black	54 (24)	48 (27)	6 (13)	
Asian	15 (7)	13 (7)	2 (4)	
Other	58 (26)	46 (26)	12 (26)	
Unknown	10 (5)	7 (4)	3 (7)	
Ethnicity, *n* (%)				
Non-Hispanic	144 (65)	116 (66)	28 (61)	0.35
Hispanic	61 (27)	45 (26)	16 (35)	
Unknown/declined	17 (8)	15 (9)	2 (4)	
Insurance status, *n* (%)				0.85
Public^2^	183 (82)	146 (83)	37 (80)	
Private	23 (10)	18 (10)	5 (11)	
None	16 (7)	12 (7)	4 (9)	
**Clinical characteristics**				
NHSN^3^ unit on day of HO-MRSA, *n* (%)				0.60
Non-ICU	133 (60)	107 (61)	26 (57)	
ICU	89 (40)	69 (39)	20 (43)	
Device present on day of HO-MRSA, *n* (%)				
Central line	92 (41)	77 (44)	15 (33)	0.17
Ventilator	63 (28)	51 (29)	12 (26)	0.70
SARS-CoV-2 PCR within 30 days, *n* (%)				0.08
Negative	159 (72)	128 (73)	31 (67)	
Positive	36 (16)	24 (14)	12 (26)	
Not performed	27 (12)	24 (14)	3 (7)	
Positive nares MRSA PCR within 1 year, *n* (%)				0.12
Negative	24 (11)	22 (13)	2 (4)	
Positive	65 (29)	54 (31)	11 (24)	
Not performed	133 (60)	100 (57)	33 (72)	
Positive MRSA culture within 1 year, *n* (%)	64 (29)	55 (31)	9 (20)	0.12

1 From 2020–2023, 222 patients were diagnosed as having HO-MRSA BSI.

2 Public insurance includes Medicare, Medicaid, Veterans Administration.

3 National Health Safety Network.

Overall, 176 (79%) were hospitalized in tertiary hospitals and 46 (21%) were hospitalized in community hospitals (Table 1). The demographic and clinical characteristics of patients with HO-MRSA BSI at tertiary and community hospitals were generally similar except for age, with a lower median age in the 5 tertiary (including a dedicated children’s hospital) (63 years, IQR 46–75) compared to 4 community (69 years, IQR 54–77) hospitals (*p* = .04). Over time, the number of patients with private insurance decreased as did the number of ICU patients (Table 2). The proportion of patients on a ventilator varied over time with the lowest number of ventilated patients occurring in 2022. Positive SARS-CoV-2 and MRSA PCR results also varied over time.

**Table 2.** Demographic and clinical characteristics of patients by year with hospital-onset methicillin-resistant *Staphylococcus aureus* bloodstream infection (HO-MRSA BSI), 2020 to 2023

	All years *n* = 224^1^	2020 *n* = 63 (28%)	2021 *n* = 59 (26%)	2022 *n* = 48 (21%)	2023 *n* = 54 (24%)	*P*-value
**Demographic characteristics**						
Age, years, median (IQR)	65 (49–76)	61 (37–73)	65 (49–72)	70 (58–77)	63 (49–78)	0.23
Age strata, years, *n* (%)						0.17
< 18	27 (12)	12 (19)	7 (12)	3 (6)	5 (9)	
18–64	84 (38)	23 (37)	22 (37)	16 (33)	23 (43)	
65–74	51 (23)	14 (22)	18 (31)	12 (25)	7 (13)	
≥ 75	62 (28)	14 (22)	12 (20)	17 (35)	19 (35)	
Sex, *n* (%)						0.83
Male	140 (63)	41 (65)	34 (58)	31 (65)	34 (63)	
Female	84 (38)	22 (35)	25 (42)	17 (35)	20 (37)	
Race, *n* (%)						0.95
White	86 (38)	22 (35)	19 (32)	20 (42)	25 (46)	
Black	54 (24)	14 (22)	18 (31)	11 (23)	11 (20)	
Asian	15 (7)	3 (5)	6 (10)	4 (8)	2 (4)	
Other	59 (26)	20 (32)	14 (24)	10 (21)	15 (28)	
Unknown	10 (4)	4 (6)	2 (3)	3 (6)	1 (2)	
Ethnicity, *n* (%)						0.98
Non-hispanic	146 (65)	39 (62)	40 (68)	32 (67)	35 (65)	
Hispanic	61 (27)	20 (32)	14 (24)	13 (27)	14 (26)	
Unknown	17 (8)	4 (6)	5 (8)	3 (6)	5 (9)	
Insurance status, *n* (%)						**0.01**
Public^2^	185 (83)	45 (71)	46 (78)	44 (92)	50 (93)	
Private	23 (10)	11 (17)	6 (10)	4 (8)	2 (4)	
None	16 (7)	7 (11)	7 (12)	0 (0)	2 (4)	
Hospital setting, *n* (%)						0.31
Tertiary	178 (79)	53 (84)	49 (83)	34 (71)	42 (78)	
Community	46 (21)	10 (16)	10 (17)	14 (29)	12 (22)	
**Clinical characteristics**						
NHSN^3^ Unit, *n* (%)						**0.04**
Non-ICU	134 (60)	30 (48)	33 (56)	34 (71)	37 (69)	
ICU	90 (40)	33 (52)	26 (44)	14 (29)	17 (31)	
Device present on day of HO-MRSA, *n* (%)						
Central line	93 (42)	33 (52)	26 (44)	16 (33)	18 (33)	0.11
Ventilator	64 (29)	24 (38)	18 (31)	6 (13)	16 (30)	**0.03**
SARS-CoV-2 PCR results within 30 days, *n* (%)						**< 0.01**
Negative	161 (72)	35 (56)	43 (73)	37 (77)	46 (85)	
Positive	36 (16)	12 (19)	13 (22)	7 (15)	4 (7)	
Not performed	27 (12)	16 (25)	3 (5)	4 (8)	4 (7)	
Nares MRSA PCR within 1 year, *n* (%)						**< 0.05**
Negative	24 (11)	12 (19)	5 (8)	2 (4)	5 (9)	
Positive	67 (30)	11 (17)	22 (37)	14 (29)	20 (37)	
Not performed	133 (59)	40 (63)	32 (54)	32 (67)	29 (54)	
Positive MRSA culture within 1 year, *n* (%)						0.23
	65 (29)	17 (27)	20 (34)	9 (19)	19 (35)	

1 From 2020–2023, 222 patients were diagnosed as having HO-MRSA BSI, with 2 patients diagnosed in more than 1 year.

2 Public insurance includes Medicare, Medicaid, Veterans Administration.

3 National Healthcare Safety Network.

### HO-MRSA BSI SIRs and rates per 10,000 patient-discharges

**Figure 1.**
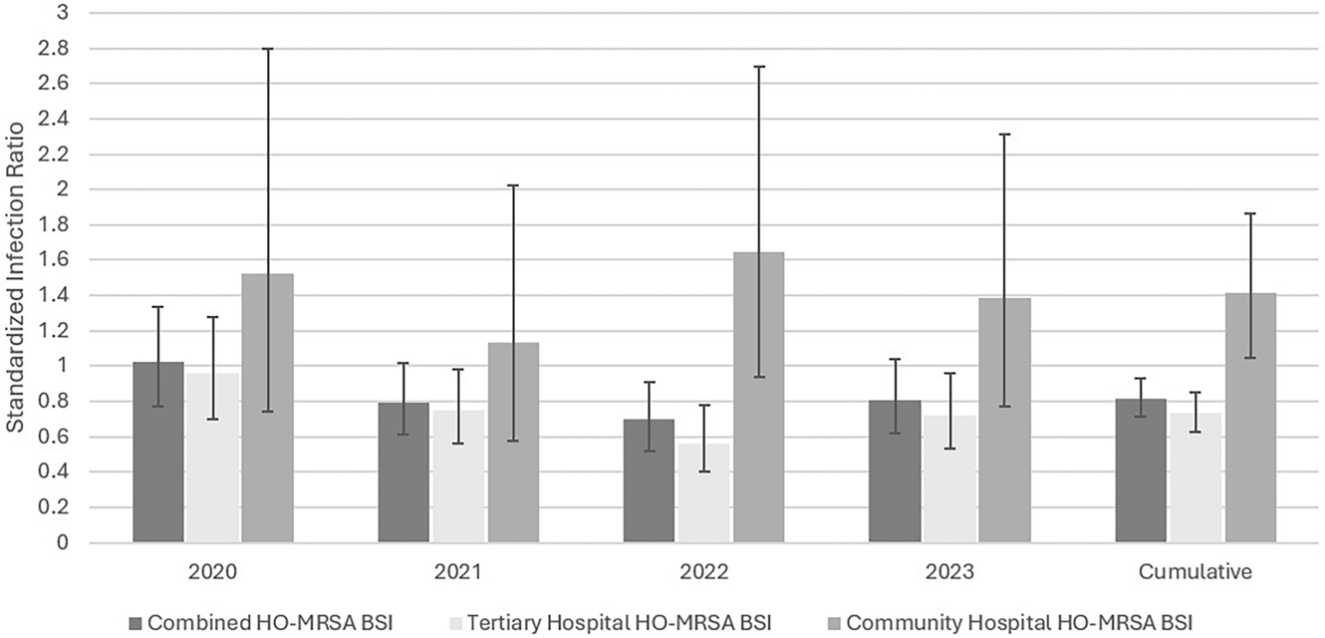
The standardized infection ratio (SIR) for hospital-onset methicillin-resistant *Staphylococcus aureus* Bloodstream Infection (HO-MRSA BSI), 2020–2023.

The combined SIRs were similar each year of the study. The SIRs for tertiary hospitals were similar each year of the study, as were the SIRs for community hospitals (Figure 1). However, the cumulative 2020–2023 SIR was higher for community hospitals (1.312) than for tertiary hospitals (0.732) (Relative ratio [RR] 1.93, 95% CI 1.38, 2.66, *p* = .0002). Similarly, the 2022 SIR was higher in community hospitals than tertiary hospitals (RR 2.92, 95% CI 1.52, 5.36, *p* = .002).

The overall rates of HO-MRSA BSI per 10,000 patient-discharges for 2020 through 2023 were 3.53, 3.10, 2.58, and 2.95, respectively, with no statistical differences between all paired comparisons (data not shown). When analyzed by setting, there was only one rate difference-2022 was lower than 2020 (2.34 vs 3.81, RR 0.61, *p* = .02) among tertiary hospitals.

### Sources of HO-MRSA BSI

Among the 232 HO-MRSA BSI episodes, 223 primary sources of infection were identified (Table 3). Fourteen episodes had more than 1 primary source. The most common source of HO-MRSA BSI was pneumonia (92/223, 41%) including non-ventilator hospital-associated pneumonia (NVHAP), ventilator-associated pneumonia (VAP), and a single case of community-associated pneumonia (CAP). Of the 48 cases due to NVHAP, 31% occurred in the ICU and the proportion did not significantly change over time. A higher proportion of patients tested positive for SARS-CoV-2 at community hospitals compared to patients at tertiary hospitals (10/19, 53% vs 15/73, 21%, respectively, *p* = .01). The second, third, and fourth most common sources of HO-MRSA BSI were skin and soft tissue infections (SSTIs) (37/223, 17%), central line-associated bloodstream infections (CLABSIs) (28/223, 13%), and peripheral intravenous (PIV) catheter-related issues (20/223, 9%). The source of HO-MRSA BSI could not be determined for 9.9% (23/232) of episodes (11% TH and 4% CH).

**Table 3.** Primary sources of hospital-onset methicillin-resistant *Staphylococcus aureus* bloodstream infection (HO-MRSA BSI) in tertiary versus community hospitals, 2020–2023

Primary sources, *n* (%)	All hospitals *n* = 223^1^	Tertiary hospitals *n* = 176 (79%)	Community hospitals *n* = 47 (21%)
Pneumonia	92 (41)	73 (41)	19 (40)
*Community-associated pneumonia*	*1/92 (1)*	*1/73 (1)*	*0/19 (0)*
*Non-ventilator hospital-associated pneumonia*	*48/92 (52)*	*39/73 (53)*	*9/19 (47)*
*Ventilator-associated pneumonia*	*43/92 (47)*	*33/73 (45)*	*10/19 (53)*
Skin and soft tissue infection	37 (17)	27 (15)	10 (21)
Central line-associated bloodstream infection	28 (13)	26 (15)	2 (4)
Peripheral line insertion site	20 (9)	13 (7)	7 (15)
Endocarditis	18 (8)	16 (9)	2 (4)
Intra-abdominal	7 (3)	4 (2)	3 (6)
Urine (no catheter-associated urinary tract infection)	6 (3)	4 (2)	2 (4)
Surgical site infection	4 (2)	4 (2)	0 (0)
Osteomyelitis	4 (2)	3 (2)	1 (2)
Other^2^	7 (3)	6 (3)	1 (2)
Unknown^3^	23 (9.9 %)	21 (11%)	2 (4%)

1 Of 232 episodes, 223 sources were known and 23 were unknown. Fourteen episodes had more than one source.

2 Other sources include arteriovenous graft site, extracorporeal mechanical oxygenation circuit, vascular graft, midline catheter, thrombophlebitis, mediastinitis, and catheter insertion site.

3 The denominator for unknown sources is based on all 232 hospitals episodes.

### Outcomes: Mortality and length of stay

The in-hospital all-cause mortality rate and in hospital 30-day all-cause mortality rate were 35% (77/222) and 18% (41/222), with the community hospitals rate higher than the tertiary hospitals (Table 4). The median number of days from HO-MRSA BSI to death was 11 days (IQR 4–25) and similar among patients at tertiary and community hospitals (11 vs 12 days, *p* = .82).

**Table 4.** Outcomes of hospital-onset methicillin-resistant *Staphylococcus aureus* bloodstream infection (HO-MRSA BSI) in tertiary versus community hospitals, 2020–2023

Outcomes	All hospitals	Tertiary hospitals	Community hospitals	*P*-value
**Per patient** ^1^	***n*** **= 222**	***n*** **= 176**	***n*** **= 46**	
All-cause mortality during hospitalization, *n* (%)	77 (35)	59 (34)	18 (39)	0.48
30-day All-cause mortality during hospitalization, *n* (%)	41 (18)	28 (16)	13 (28)	0.05
Days from HO-MRSA to death, median days, (IQR)^2^	11 (4–25)	11 (4–26)	12 (2–17)	0.82
**Per first episode**	***n*** **= 222**	***n*** **= 176**	***n*** **= 46**	
Days from admission to HO-MRSA, median days (IQR)	12 (7–25)	15 (7–29)	9 (6–16)	**< 0.01**
Length of stay, median days (IQR)	31 (19–62)	38 (21–66)	24 (16–35)	**< 0.01**

1 In patients with more than one episode, 30-day crude mortality and median days from HO-MRSA to death determined from second (*n* = 8 patients) or third (*n* = 1 patient) HO-MRSA episode only. If patient had more than one episode, data from last episode used.

2 Denominator *n* = 119 for all hospitals, *n* = 102 for tertiary hospitals, and *n* = 17 for community hospitals of patients with length of stay ≥ 30 calendar days.

Among the 222 first episodes of HO-MRSA BSI, the median number of days from admission until HO-MRSA BSI was 12 days and was significantly longer for episodes that occurred in tertiary hospitals compared to community hospitals (15 vs 9 median days, respectively, *p* < .01) (Table 4). The median total length of stay was 31 days and was longer for patients at tertiary hospitals than those at community hospitals (38 vs 24 median days, respectively, *p* < .01).

## Discussion

In a multicenter study encompassing tertiary and community hospitals, we identified 222 patients with HO-MRSA BSI who had 232 episodes over a 4-year study period. Fewer than half were in ICUs (40%), 41% had central lines and 28% were mechanically ventilated. The combined SIR was similar during each study year, although the cumulative SIR (combining SIRs from 2020–2023) for community hospitals was higher than that of the tertiary hospitals. Pneumonia, followed by SSTIs, CLABSIs, and PIV catheter-related issues were the most common sources associated with HO-MRSA BSI. These surveillance findings are informative when considering future interventions.

Although 80% of HO-MRSA BSI episodes occurred in tertiary care hospitals, patients in community hospitals contributed to the overall burden of this healthcare-associated infection (HAI) and in fact had a higher cumulative SIR. We found that the demographic characteristics of patients hospitalized in tertiary versus community hospitals were similar except that those in tertiary care hospitals had a younger median age, which could be explained by inclusion of a hospital with only pediatric patients. Similarly, the most common primary sources of infection in both types of hospitals were pneumonia and SSTIs. The proportion of patients with device use and SARS-CoV-2 infections were also similar at tertiary and community hospitals. Thus, attention to hospitals setting may be important when considering feasibility of implementing interventions.

The HO-MRSA BSI SIRs and rate were unchanged during the 4 years of this study which started during the COVID-19 pandemic. Like other HAIs that increased during the COVID-19 pandemic,^8^ HO-MRSA BSI peaked nationally and then returned to pre-pandemic levels.^9^ Our findings may be different because New York City had a high initial burden of COVID-19 infections and our annual number of HO-MRSA BSI cases might be too small to see similar trends to those observed nationally. Patients in tertiary hospitals had a longer length of hospitalization prior to HO-MRSA BSI and a longer length of overall stay likely explained by the increased acuity of patients at tertiary hospitals. We found a 31 day median overall length of hospital stay for patients with HO-MRSA BSI, consistent with the prior literature which ranged from 16–35 days.^10,11^ We also found a 35% overall all-cause mortality rate, similar to previous reports in the literature of 36%–38.1%,^10,11^ with death occurring a median of 11 days after HO-MRSA BSI. These findings confirm that HO-MRSA BSI is associated with high rates of morbidity and mortality and further highlight the need for effective preventive strategies for this HAI.

In the current study, pneumonia, SSTIs, CLABSIs, and PIV catheter-related issues were the most common sources for HO-MRSA BSI, but few studies have examined the primary sources of infection leading to this entity. Studies of all-cause HOB and fungemia caused by non-commensal organisms have noted that gastrointestinal (including neutropenic translocation) sources; endovascular sources, including CLABSIs and PIV catheters; and device-associated infections, including urinary catheters and ventilators are the most common.^12–14^ Although pneumonia was very common in our study, it was not a common source in these studies of all-cause HOB with rates of 4.1%–12%, pneumonia was also uncommon in MRSA-specific studies. In one study among patients with community- or hospital-onset MRSA pneumonia, 12.2% of patients had concurrent bacteremia,^15^ and in another study among patients with MRSA BSI, 11% had concurrent pneumonia.^11^ Less is known about SSTIs as primary sources of HO-MRSA BSI with one study reporting that 21% of cases were due to SSTIs.^14^

Our findings confirm the importance of attributable sources of HO-MRSA BSI to inform prevention strategies. The high rate of the primary source of pneumonia suggests that emphasis on strategies to prevent pneumonia, for example, enhanced oral care with tooth brushing and elevation of the head of the bed to 30^º^, could reduce pneumonia and associated BSI.^16^ However, the interventions of CHG bathing and intranasal mupirocin recommended for ICU patients^4^ may be insufficient to prevent NVHAP cases, as many patients with NVHAP-associated MRSA BSI were not in the ICU and therefore would not have received ICU-based decolonization. Thus, enhanced oral care and general pneumonia prevention strategies^16^ need to be emphasized in both ICU and non-ICU settings. Similarly, ensuring evidence-based best practices for PIV catheter insertion and maintenance, for example, hand hygiene and aseptic technique, could reduce the risk of BSI.^17–19^

Furthermore, the high rate of colonization with MRSA prior to HO-MRSA BSI supports active MRSA decolonization strategies.^4,20^ Consideration of expanding decolonization efforts beyond the ICU population^4^ may be warranted as 60% of patients in the current study were not hospitalized in ICUs.

### Limitations

This study had several limitations. Although our study included a large urban multicampus healthcare system, our findings may not be generalizable to other hospital or geographic settings. During the early years of the study period, hospitals sequentially transitioned to the Epic^®^ EMR which could have led to missed HO-MRSA BSI episodes for study inclusion. Misattribution of cases to ICUs or to non-ICU may occur if patients are transferred between units. Our study design did not permit an analysis of demographic factors associated with HO-MRSA BSI. Primary infectious sources could have been misclassified due to the limitations of retrospective review, missing data, and use of clinical definitions for some sources, such as pneumonia and SSTI. Additionally, PCR testing and decolonization for MRSA were not standardized. Lastly, all-cause mortality measures represented overall and not attributable mortality and could be over-estimated as we only included the last HO-MRSA BSI episode for those with more than one episode who died.

## Conclusions

The unchanged HO-MRSA BSI SIRs and rates per 10,000 patient-discharges determined in this study support the need for additional interventions that focus on prevention of the primary sources of MRSA infections. Ongoing systematic surveillance of the primary sources of HO-MRSA BSI should be incorporated to inform, implement, and monitor best practices for prevention.

## Supporting information

Singh et al. supplementary material Singh et al. supplementary material

## Supplementary material

The supplementary material for this article can be found at https://doi.org/10.1017/ice.2025.10370.

## References

[1] Centers for Disease Control and Prevention. Centers for Medicaid and Medicare Services Operational Guidance for Acute Care Hospitals to Report FACWIDEIN MRSA LabId Event Data, 2019. https://www.cdc.gov/nhsn/pdfs/cms/final-ach-mrsa-bacteremia-guidance.pdf. Accessed May 2025.

[2] National Healthcare Safety Network (NHSN). Patient Safety Component Manual, 2024. https://www.cdc.gov/nhsn/pdfs/pscmanual/pcsmanual_current.pdf. Accessed December 2024.

[3] National Healthcare Safety Network (NHSN) Bloodstream Infection Event (Central Line-Associated Bloodstream Infection and Non-central Line Associated Bloodstream Infection), 2024. https://www.cdc.gov/nhsn/pdfs/pscmanual/pcsmanual_current.pdf. Accessed December 2024. https://www.cdc.gov/nhsn/pdfs/pscmanual/pcsmanual_current.pdf, 2024.

[4] Popovich KJ, Aureden K, Ham DC, et al. SHEA/IDSA/APIC practice recommendation: Strategies to prevent methicillin-resistant Staphylococcus aureus transmission and infection in acute-care hospitals: 2022 update. Infect Control Hosp Epidemiol 2023;44:1039–1067. 37381690 10.1017/ice.2023.102 PMC10369222

[5] Centers for Disease Control and Prevention. HAI Pathogens and Antimicrobial Resistance Report: 2018-2021. https://www.cdc.gov/nhsn/hai-report/index.html. Accessed May 2025.

[6] Centers for Disease Control and Prevention. The NHSN Standardized Infection Ratio (SIR). https://www.cdc.gov/nhsn/pdfs/ps-analysis-resources/nhsn-sir-guide.pdf. Accessed March 2025.

[7] Centers for Disease Control and Prevention. National Health Services Network. Patient Safety Analysis Resources. https://www.cdc.gov/nhsn/pdfs/ps-analysis-resources/statscalc.pdf. Accessed August 2025.

[8] Centers for Disease Control and Prevention. COVID-19 Impact on Hospital Acquired Infections, 2024. https://www.cdc.gov/healthcare-associated-infections/php/data/covid-impact.html. Accessed May 2025.

[9] Biggs HM, Li R, Jackson KA, et al. Trends in incidence and epidemiology of methicillin-resistant Staphylococcus aureus bacteremia, six emerging infections program surveillance sites, 2005-2022. Open Forum Infect Dis 2025;12:ofaf282. 40453873 10.1093/ofid/ofaf282 PMC12125674

[10] Tsuzuki S, Yu J, Matsunaga N, Ohmagari N. Length of stay, hospitalisation costs and in-hospital mortality of methicillin-susceptible and methicillin-resistant Staphylococcus aureus bacteremia in Japan. Public Health 2021;198:292–296. 34507134 10.1016/j.puhe.2021.07.046

[11] de Kraker ME, Wolkewitz M, Davey PG, et al. Clinical impact of antimicrobial resistance in European hospitals: Excess mortality and length of hospital stay related to methicillin-resistant Staphylococcus aureus bloodstream infections. Antimicrob Agents Chemother 2011;55:1598–1605. 21220533 10.1128/AAC.01157-10 PMC3067153

[12] Leekha S, Robinson GL, Jacob JT, et al. Evaluation of hospital-onset bacteraemia and fungaemia in the USA as a potential healthcare quality measure: A cross-sectional study. BMJ Qual Saf 2024;33:487–498. 10.1136/bmjqs-2023-016831 PMC11287649 38782579

[13] Gandra S, Singh SK, Chakravarthy M, et al. Epidemiology and preventability of hospital-onset bacteremia and fungemia in 2 hospitals in India. Infect Control Hosp Epidemiol 2024;45:157–166. 37593953 10.1017/ice.2023.170 PMC10877540

[14] Stack MA, Dbeibo L, Fadel W, Kelley K, Sadowski J, Beeler C. Etiology and utility of hospital-onset bacteremia as a safety metric for targeted harm reduction. Am J Infect Control 2024;52:195–199. 37295676 10.1016/j.ajic.2023.06.002

[15] Shorr AF, Zilberberg MD, Micek ST, Kollef MH. Outcomes associated with bacteremia in the setting of methicillin-resistant Staphylococcus aureus pneumonia: A retrospective cohort study. Crit Care 2015;19:312. 26335247 10.1186/s13054-015-1029-z PMC4558880

[16] Klompas M, Branson R, Cawcutt K, et al. Strategies to prevent ventilator-associated pneumonia, ventilator-associated events, and nonventilator hospital-acquired pneumonia in acute-care hospitals: 2022 update. Infect Control Hosp Epidemiol 2022;43:687–713. 35589091 10.1017/ice.2022.88 PMC10903147

[17] Zingg W, Barton A, Bitmead J, et al. Best practice in the use of peripheral venous catheters: A scoping review and expert consensus. Infect Prev Pract 2023;5:100271. 36910422 10.1016/j.infpip.2023.100271 PMC9995289

[18] Thompson J, Steinheiser MM, Hotchkiss JB, et al. Standards of care for peripheral intravenous catheters: Evidence-based expert consensus. Br J Nurs 2024;33:S32–S46. 10.12968/bjon.2024.0422 39585227

[19] Zanella MC, Catho G, Jackson H, et al. Dwell time and risk of bloodstream infection with peripheral intravenous catheters. JAMA Netw Open 2025;8:e257202. 40272799 10.1001/jamanetworkopen.2025.7202 PMC12022809

[20] Huang SS, Septimus E, Kleinman K, et al. Targeted versus universal decolonization to prevent ICU infection. N Engl J Med 2013;368:2255–2265. 23718152 10.1056/NEJMoa1207290 PMC10853913

