# Supplementary material for: Hospital-onset methicillin-resistant Staphylococcus aureus bloodstream infections within tertiary and community hospitals and implications for prevention
Source: Infect Control Hosp Epidemiol. 2025 Dec 9;47(3):236–43. doi: 10.1017/ice.2025.10370 (PMC12932923; doi:10.1017/ice.2025.10370)
Supplement: Singh et al. supplementary material [file S0899823X2510370Xsup001.docx]

**Supplemental Table 1:** Demographic and Clinical Characteristics of Patients by Year with Hospital-Onset Methicillin-Resistant *Staphylococcus aureus* Bloodstream Infection (HO-MRSA BSI)*,* 2020 to 2023.

|  | **All years**  n = 224 | **2020**  n = 63 (28%) | **2021**  n = 59 (26%) | **2022**  n = 48 (21%) | **2023**  n = 54 (24%) | **P-value** |
| --- | --- | --- | --- | --- | --- | --- |
| **Demographic Characteristics** | | | | | | |
| Age, years, median (IQR) | 65 (49-76) | 61 (37-73) | 65 (49-72) | 70 (58-77) | 63 (49-78) | 0.23 |
| Age strata, years, n (%) |  |  |  |  |  | 0.17 |
| < 18 | 27 (12) | 12 (19) | 7 (12) | 3 (6) | 5 (9) |  |
| 18-64 | 84 (38) | 23 (37) | 22 (37) | 16 (33) | 23 (43) |  |
| 65-74 | 51 (23) | 14 (22) | 18 (31) | 12 (25) | 7 (13) |  |
| > 75 | 62 (28) | 14 (22) | 12 (20) | 17 (35) | 19 (35) |  |
| Sex, n (%) |  |  |  |  |  | 0.83 |
| Male | 140 (63) | 41 (65) | 34 (58) | 31 (65) | 34 (63) |  |
| Female | 84 (38) | 22 (35) | 25 (42) | 17 (35) | 20 (37) |  |
| Race, n (%) |  |  |  |  |  | 0.95 |
| White | 86 (38) | 22 (35) | 19 (32) | 20 (42) | 25 (46) |  |
| Black | 54 (24) | 14 (22) | 18 (31) | 11 (23) | 11 (20) |  |
| Asian | 15 (7) | 3 (5) | 6 (10) | 4 (8) | 2 (4) |  |
| Other | 59 (26) | 20 (32) | 14 (24) | 10 (21) | 15 (28) |  |
| Unknown | 10 (4) | 4 (6) | 2 (3) | 3 (6) | 1 (2) |  |
| Ethnicity, n (%) |  |  |  |  |  | 0.98 |
| Non-Hispanic | 146 (65) | 39 (62) | 40 (68) | 32 (67) | 35 (65) |  |
| Hispanic | 61 (27) | 20 (32) | 14 (24) | 13 (27) | 14 (26) |  |
| Unknown | 17 (8) | 4 (6) | 5 (8) | 3 (6) | 5 (9) |  |
| Insurance Status, n (%) |  |  |  |  |  | **0.01** |
| Public^1^ | 185 (83) | 45 (71) | 46 (78) | 44 (92) | 50 (93) |  |
| Private | 23 (10) | 11 (17) | 6 (10) | 4 (8) | 2 (4) |  |
| None | 16 (7) | 7 (11) | 7 (12) | 0 (0) | 2 (4) |  |
| **Clinical Characteristics** | | | | | | |
| NHSN^2^ Unit**,** n (%) | | | | | | **0.04** |
| Non-ICU | 134 (60) | 30 (48) | 33 (56) | 34 (71) | 37 (69) |  |
| ICU | 90 (40) | 33 (52) | 26 (44) | 14 (29) | 17 (31) |  |
| Device present on day of HO-MRSA, n (%) | | | | | |  |
| Central Line | 93 (42) | 33 (52) | 26 (44) | 16 (33) | 18 (33) | 0.11 |
| Ventilator | 64 (29) | 24 (38) | 18 (31) | 6 (13) | 16 (30) | **0.03** |
| SARS-CoV-2 PCR results within 30 days, n (%) | | | | | | **<0.01** |
| Negative | 161 (72) | 35 (56) | 43 (73) | 37 (77) | 46 (85) |  |
| Positive | 36 (16) | 12 (19) | 13 (22) | 7 (15) | 4 (7) |  |
| Not Performed | 27 (12) | 16 (25) | 3 (5) | 4 (8) | 4 (7) |  |
| Nares MRSA PCR within 1 year, n (%) | | | |  |  | **<0.05** |
| Negative | 24 (11) | 12 (19) | 5 (8) | 2 (4) | 5 (9) |  |
| Positive | 67 (30) | 11 (17) | 22 (37) | 14 (29) | 20 (37) |  |
| Not Performed | 133 (59) | 40 (63) | 32 (54) | 32 (67) | 29 (54) |  |
| Positive MRSA culture within 1 year, n (%) | | | | | | 0.23 |
|  | 65 (29) | 17 (27) | 20 (34) | 9 (19) | 19 (35) |  |

^1^Public includes Medicare, Medicaid, Veterans Administration. ^2^National Healthcare Safety Network Uni
